# Genome editing of pseudorabies virus in the CRISPR/Cas9 era: a mini-review

**DOI:** 10.3389/fvets.2023.1237186

**Published:** 2023-07-05

**Authors:** Hai-Ming Wang, Yang-Yang Qiao, Bing-Yan Cai, Ju Tan, Lei Na, Yu Wang, Hui Lu, Yan-Dong Tang

**Affiliations:** ^1^Jiangsu Agri-animal Husbandry Vocational College, Taizhou, Jiangsu, China; ^2^State Key Laboratory for Animal Disease Control and Prevention, Harbin Veterinary Research Institute of Chinese Academy of Agricultural Sciences, Harbin, China; ^3^Jiangsu Vocational College Agriculture and Forestry, Taizhou, Jiangsu, China; ^4^Heilongjiang Provincial Research Center for Veterinary Biomedicine, Harbin, China

**Keywords:** CRISPR/Cas9, pseudorabies virus, editing, knock out, knock in

## Abstract

Pseudorabies virus (PRV) is an important swine virus that has a significant impact on the global swine industry. PRV is a member of the herpesvirus family, specifically the alphaherpesvirus subfamily, and has been extensively utilized as a prototype herpesvirus. Notably, recent studies have reported that PRV sporadically spills over into humans. The PRV genome is approximately 150 kb in size and is difficult to manipulate at the genomic level. The development of clustered regularly interspaced short palindromic repeat-associated protein (CRISPR/Cas9) technology has revolutionized PRV genome editing. CRISPR/Cas9 has been widely used in the construction of reporter viruses, knock-out/knock-in of genes of interest, single virus tracking and antiviral strategies. Most importantly, for vaccine development, virulence gene knockout PRV vaccine candidates can be obtained within 2 weeks using CRISPR/Cas9. In this mini-review, we provide a concise overview of the application of CRISPR/Cas9 in PRV research and mainly share our experience with methods for efficiently editing the PRV genome. Through this review, we hope to give researchers better insight into the genome editing of pseudorabies virus.

## Introduction

The swine industry suffers significant financial losses worldwide due to the presence of pseudorabies virus (PRV), which is a crucial pathogen for pigs (1, 2). PRV is a highly contagious virus that affects the nervous system of pigs, leading to neurological symptoms, such as paralysis and death (1, 2). The virus is highly contagious and can spread rapidly among pigs through direct contact or through contact with contaminated objects. Moreover, there has been a concerning trend regarding the increased ability of PRV to transmit across different species, as evidenced by outbreaks of PRV variants. This poses a significant risk to humans, a fact that has been well documented in several recent reviews (3–5). PRV belongs to the alphaherpesvirus group, which is closely related to herpes simplex virus-1 (HSV-1) and varicella-zoster virus (VZV). These viruses are known for their neurotropism and ability to establish lifelong latency in their natural hosts (6, 7). Consequently, PRV is frequently utilized as a model to understand the molecular details of alphaherpesviruses and examine the functions of the nervous system in mammals (8, 9).

Due to its large genome, which contains more than 70 genes with varying functions, the genomic manipulation of PRV was a major challenge in herpesvirus research prior to the emergence of CRISPR technology (10). In early studies, the genome of herpesviruses was manipulated by subjecting the infected cells to a range of physical, chemical, or biological conditions (11, 12), but the resulting mutation is not site-specific and is often randomly scattered throughout the whole genome. Furthermore, the mutation frequency is extremely low, and acquiring interesting mutations is always time-consuming and labor-intensive (11, 12). When the recombination strategy was introduced to manipulate the herpesvirus genome, precise manipulation of the desired specific gene became possible. By transfecting an interesting DNA fragment with homologous arms into infected cells or cotransfecting it with viral genomic DNA, the homologous DNA fragment recombined with the target herpesvirus genome. However, the efficiency of homologous recombination using these methods was extremely low (ranging from 1 in 10^6^ to 1 in 10^7^). The development of bacterial artificial chromosome (BAC) technology has revolutionized the genetics of herpesviruses (11–15). Advances in BAC-based genome editing have been instrumental in helping us gain insight into herpesvirus gene function and vaccine development, thus furthering our knowledge of herpesviruses and paving the way for more effective vaccines and treatments (this is well reviewed by Xia and coworkers (16)). Despite its advantages, BAC-based mutagenesis has some drawbacks. For instance, it can only be used after an infectious BAC has been created, and in some cases, the BAC vector must be removed from herpesviruses (10, 17, 18).

The emergence of genome editing technologies, specifically programmable nucleases such as zinc finger nucleases (ZFNs), transcription activator-like effector nucleases (TALENs), and the CRISPR/Cas9 RNA-guided endonuclease system, has opened up a wide range of possibilities for their use in various life science applications (19–21). In regard to editing at specific sites, ZFNs and TALENs use DNA-binding proteins and the FokI nuclease domain, whereas CRISPR/Cas9 utilizes guide RNAs and the Cas9 protein. The fundamental concept behind these technologies is to cut DNA in a site-specific manner, which generates double-strand breaks (DSBs) at targeted sites. DSBs then stimulate the activation of endogenous DNA repair systems, which can lead to targeted genome modification through either homology-directed repair (HDR) or error-prone nonhomologous end joining (NHEJ). In this mini-review, we focus on the application of CRISPR/Cas9 technology in studies of PRV and mainly concentrate on strategies to efficiently edit the PRV genome.

## CRISPR/Cas9 technology and its application in PRV

As a versatile genetic modification tool, CRISPR/Cas9 has emerged as a valuable tool for genetic engineering in a variety of organisms (22, 23). CRISPR–Cas systems have been discovered in numerous bacterial and archaeal organisms, which use these systems as a means of protecting themselves against mobile genetic elements, which utilize the RNA-guided Cas9 nuclease to selectively target and cleave specific foreign DNA sequences (24, 25). The CRISPR–Cas9 system offers a simple and efficient method for manipulating cells in diverse organisms, including those relevant to medicine, agriculture, and scientific investigation. This approach is applicable to virtually all cell types, which makes it a versatile tool for researchers in various fields (24, 25). The mechanism of DNA editing by CRISPR/Cas9 involves creating DSBs in the targeted DNA, which then triggers the activation of cellular repair pathways such as NHEJ and HDR. Knock-out and knock-in of genes of interest can be achieved by utilizing both repair pathways. In comparison to BAC and homologous recombination (HR) methods, CRISPR/Cas9 presents more advantages for the editing of DNA viruses because it only requires the design of effective single-guide RNA (sgRNA) (26).

Large-genome DNA viruses, including adenovirus (27), herpes simplex virus 1 (17, 27–29), and Epstein–Barr virus (30–32), have been manipulated using the CRISPR/Cas9 system. In fact, CRISPR/Cas9 for PRV editing was first conducted by Xu et al. (33). The application of CRISPR/Cas9 in PRV includes the construction of reporter viruses (34–38), vaccine development (39–47), the exploration of virulence genes (48, 49), the studying of viral protein function (49–56), single virus tracking (57), and the development of CRISPR/Cas9-based antiviral strategies (58, 59).

## Improving PRV editing efficacy by CRISPR/Cas9

### sgRNA design

In CRISPR/Cas9 editing, effective sgRNA is critical for successful editing. Many software and online tools can be utilized to predict the effectiveness and suitability of sgRNA, but the predictive power of these computing tools is not sufficient. Therefore, it is essential to conduct reliable systematic testing of the cleavage efficiency of sgRNA and Cas9. There are several methods to measure the efficacy of sgRNA. In our previous studies, we used the px330 plasmid, which harbors both sgRNA and the Cas9 expression cassette simultaneously. First, an effective sgRNA could cleave viral DNA efficiently and then inhibit the replication of PRV. We first transfected designed potential sgRNAs into cells and then infected the transfected cells 24 h later at a lower multiplicity of infection (MOI). The viral titer was then quantified to identify the most effective sgRNA. The lower MOI (always lower than 0.01) is important because the inhibitory effect of sgRNA may be limited due to the efficacy of sgRNA-mediated cleavage or transfection. When a high MOI is used, it is difficult to differentiate between effective and noneffective sgRNAs. Second, a reporter virus with either EGFP or firefly luciferase is used to evaluate the effectiveness of the sgRNA. This approach is straightforward and suitable for large-scale, high-throughput screening, and it is also cost-effective and can be implemented with minimal resources. Third, we can cotransfect tested sgRNA with the plasmid that eukaryotically expresses the target gene into HEK293T cells and then detect target gene expression by Western blot or immunofluorescence assays to select an effective sgRNA.

### Transfection-infection-based editing

**Figure 1:**
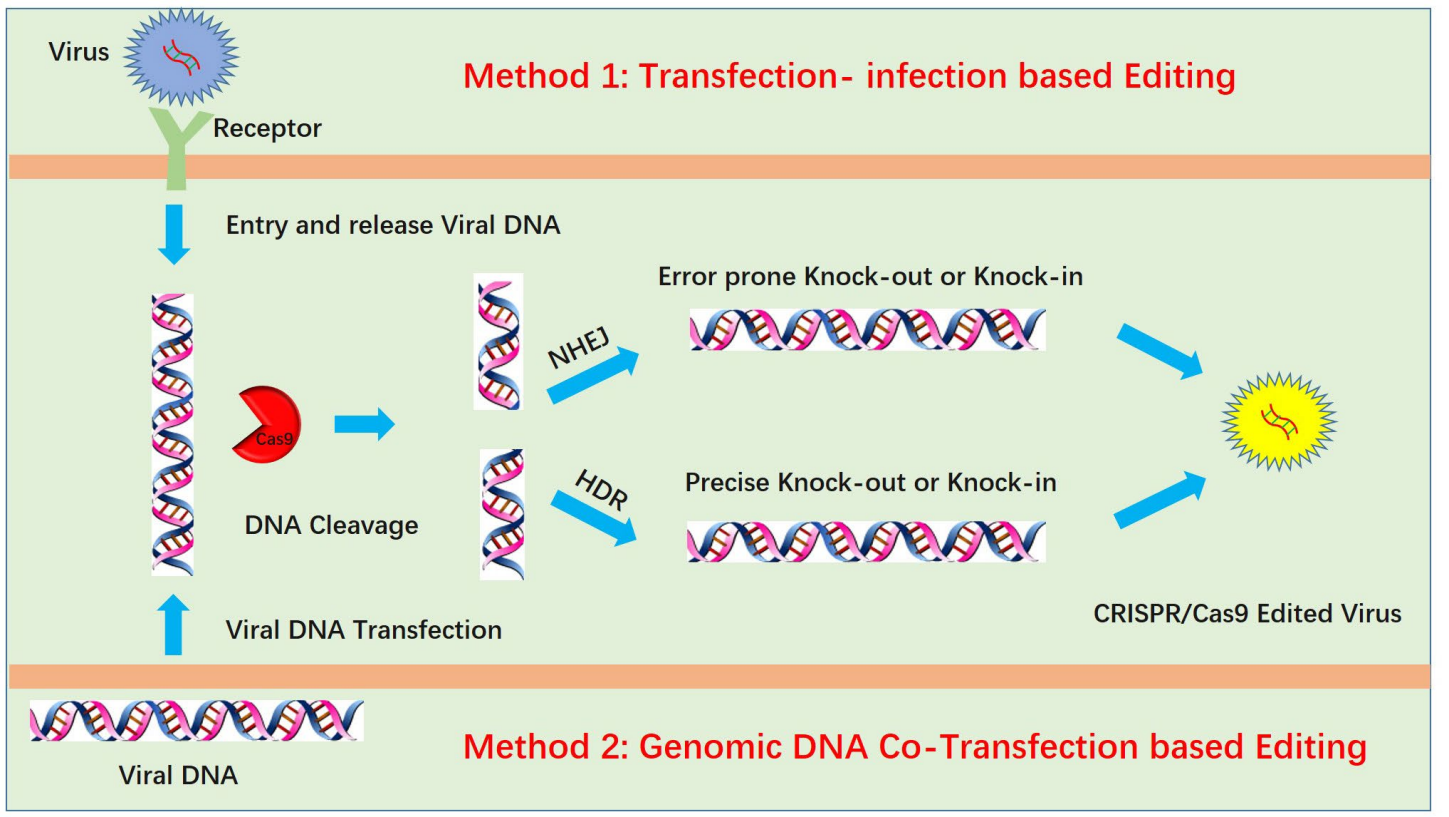
Two methods for PRV editing using CRISPR/Cas9 technology. One method is the transfection-infection-based method, which requires transfection of the CRISPR/Cas9 plasmid followed by infection of cells with PRV. Another method is the cotransfection method, which requires extraction of the high-quality intact PRV genome and cotransfection of PRV genomic DNA with the CRISPR/Cas9 plasmid. In both methods, viral DNA is cleaved by CRISPR/Cas9, and the cleaved DNA is repaired by nonhomologous end joining (NHEJ) or homology-directed repair (HDR).

Genome editing of pseudorabies virus is mainly achieved by two methods: transfection-infection-based editing and viral genomic DNA cotransfection-based editing (Figure 1). For transfection-infection-based editing, transfected cell lines should have high transfection efficacy, which could increase the opportunity for the coexistence of sgRNA and virus and maximize the probability of virus editing. Transfection of plasmids with sgRNA and Cas9 into HEK293T cells is better than transfection into Vero cells due to the high transfection efficacy of HEK293T cells. Twenty-four hours post transfection, a lower MOI (always lower than 0.01) is used to infect the transfected cells. According to our experience, a lower MOI is critical for observing the PRV-induced cytopathic effect (CPE), and a lower dose of PRV infection requires multiple cycles of replication. We speculated that this increases the likelihood of the coexistence of PRV and the CRISPR system during multiple rounds of replication. However, infection at a lower MOI is only suitable for PRV knockout mediated by the NHEJ repair pathway. For PRV recombination-mediated HDR, infection at a higher MOI increases the recombination rate (26). We hypothesize that this is due to a higher chance of coexistence between the viral DNA and donor plasmid when a higher MOI is used, which in turn increases the homologous recombination (HR) rate. Additionally, high-MOI infection always produces fewer viruses, which also increases the successful HR rate. We attribute this to the strong CPE of PRV, which kills infected cells rapidly, preventing them from completing the full viral life cycle and therefore producing fewer viruses. Notably, for infection at a higher MOI, the maximum recombination efficiency was only approximately 0.09% in our previous work (60). This finding indicated that transfection-infection-based editing may not be suitable for HR. The next step is plaque purification, which is also very important. Generally, wild-type PRV replicates faster than knock-out viruses and generates large plaques, whereas the knock-out virus produces smaller plaques. Plaque purification in 10-cm^2^ dishes may be better than that in 6-well plates because large dishes allow easy separation of plaques from each other. If plaques are easily separated, only one round of purification is enough. When a single plaque is purified and amplified in a 12-well plate, we only need to identify the virus by Western blotting at the protein level or DNA sequencing at the DNA level.

### Genomic DNA cotransfection-based editing

**Figure 2:**
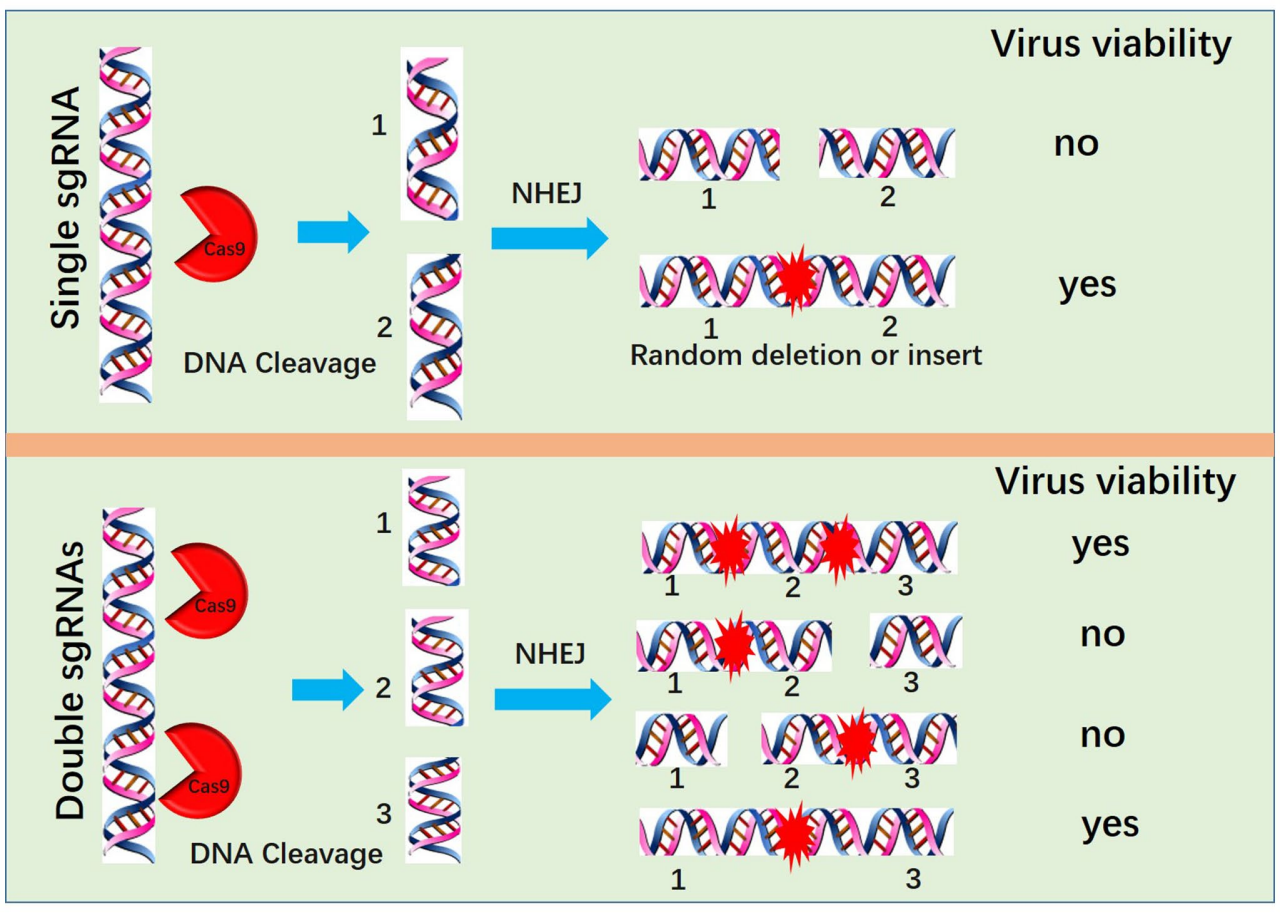
Mechanism by which two sgRNAs generate 100% knockout PRV. Two sgRNAs could break DNA into three fragments, and only when all three or two fragments, excluding the middle nonessential gene fragment, are ligated together can the virus survive; no other connection of fragments lead to a reproductive virus.

Another method for PRV editing is genomic DNA cotransfection-based editing. This method requires the extraction of high-quality and intact viral genomes followed by cotransfection with specific sgRNAs (Figure 1). For gene knock-out or knock-in, the cotransfection-based method significantly increases the editing efficacy. In our previous studies, by utilizing a transfection-infection-based method, we knocked out several PRV genes, and a single sgRNA enabled us to achieve a knock-out rate ranging from 12.5 to 42.9% (39, 48). However, when we used a cotransfection-based method, the knock-out rate of a single sgRNA reached 90.91% (26). A cotransfection assay can be used to introduce both the CRISPR system and the viral genome into the same cells, leading to improved PRV editing. However, when two sgRNAs were used, the ratio of nonessential gene knock-out reached 100%. We have proposed a model to explain why two sgRNAs could produce 100% knockout in our previous study (26). Generally, two sgRNAs could break DNA into three fragments, and only when all three or two fragments, excluding the middle nonessential gene fragment, were ligated together could the virus survive; any other connections of fragments did not lead to a reproductive virus. The chances of the fragments connecting in the same way as the original virus were quite low; thus, we obtained 100% knock-out (Figure 2). Furthermore, two sgRNAs also significantly promoted HDR-mediated knock-in efficacy. For a single sgRNA, the highest knock-in efficiency reached 40%, whereas two sgRNAs yielded the highest knock-in efficiency of up to 86% (26). The use of two sgRNAs resulted in a high knock-in efficiency, which is attributed to a reduction in background viruses. Furthermore, the replication kinetics of background viruses directly impacted the HR efficacy. A faster replication of background viruses is associated with a lower HR efficacy.

## Conclusion and future outlook

This mini-review describes how PRV may be edited efficiently by CRISPR/Cas9 and provides some insights for PRV researchers. In summary, we recommend using a genomic DNA cotransfection-based method and optimizing the use of two sgRNAs for knock out or knock in. However, CRISPR/Cas9 also has limitations, such as difficulty in single-base editing, whereas the BAC system can efficiently achieve single-base editing (61, 62). In future PRV research, a variety of genome editing tools should be employed; for example, the combination of single-base editing and CRISPR mediates knockdown (63).

## Author contributions

Y-DT and H-MW conceptualized the study and generated the figures. All authors contributed to the article and approved the submitted version.

## Funding

This study was supported by the grants from Jiangsu Agri-animal Husbandry Vocational College (NSF2023CB17).

## Conflict of interest

The authors declare that the research was conducted in the absence of any commercial or financial relationships that could be construed as a potential conflict of interest.

## Publisher’s note

All claims expressed in this article are solely those of the authors and do not necessarily represent those of their affiliated organizations, or those of the publisher, the editors and the reviewers. Any product that may be evaluated in this article, or claim that may be made by its manufacturer, is not guaranteed or endorsed by the publisher.
